# Status quo of *Experimental Physiology* – Anno 2025: What's in a name?

**DOI:** 10.1113/EP092847

**Published:** 2025-05-05

**Authors:** Damian M. Bailey, David C. Poole, Ronan M. G. Berg

**Affiliations:** ^1^ Neurovascular Research Laboratory, Faculty of Life Sciences and Education University of South Wales Pontypridd UK; ^2^ Departments of Kinesiology and Anatomy & Physiology Kansas State University Manhattan Kansas USA; ^3^ Centre for Physical Activity Research Copenhagen University Hospital–Rigshospitalet Copenhagen Denmark; ^4^ Department of Clinical Physiology and Nuclear Medicine Copenhagen University Hospital–Rigshospitalet Copenhagen Denmark; ^5^ Department of Clinical Medicine, Faculty of Health and Medical Sciences University of Copenhagen Copenhagen Denmark

As the current Editor‐in‐Chief (EiC) and Deputy Editors‐in‐Chief (USA and Europe) of *Experimental Physiology*, we have completed our three‐year tenures and take an opportunity to take a breath and reflect on the status quo of the journal. We continue to engage with our respective roles during an exciting albeit challenging period, as the publishing landscape continues to evolve rapidly, and as our journal transitioned to Open Access (Bailey, 2022; Bailey & Stewart, 2022). As we continue to navigate these waters, we remain cognisant of the challenges that loom large. How do we ensure that *Experimental Physiology* remains relevant as one of the flagship journals of The Physiological Society, while maintaining a consistently high standard of submissions and tackling issues of accessibility, affordability and equity that have long blighted scholarly communication?

At times, authors and even referees question how the broad arena of our discipline aligns with the seemingly narrower focused title of our journal – does the term ‘experimental physiology’ imply that the journal's focus should be strictly laboratory‐based? One could simply respond by quoting William Shakespeare (1564–1616), who, in *Romeo and Juliet* (Act II, Scene II), sees Juliet Capulet muse: ‘What's in a name? That which we call a rose by any other name would smell as sweet.’ However, taking a physiological rather than purely philosophical or poetic stance, we take a brief opportunity to revisit the journal's historical roots, including the conception of its title, demonstrating that the term ‘experimental’ does not refer to a specific setting, model or method, but rather reflects a fundamental principle of how physiology is practised as a science.

As previously discussed*, Experimental Physiology* was founded in 1908 by Edward A. Schäfer (1850–1935) (Figure 1), based at the University of Edinburgh (Bailey, Berg et al., 2023). Shortly after the death of his eldest son, John Sharpey Schäfer in 1918 (H.M.S. ‘Gaillardia’ struck a mine and exploded in the North Sea), whom – like all his children – he had named after his mentor and friend, William Sharpey (1802–1880), he chose to hyphenate his middle name, becoming Sharpey‐Schafer. This change served both as a tribute to his late son and as a means of honouring his mentor. At the same time, he also removed the umlaut (ä) from ‘Schäfer’, given the growing unpopularity of Germanic names in Britain following the First World War.

**FIGURE 1:**
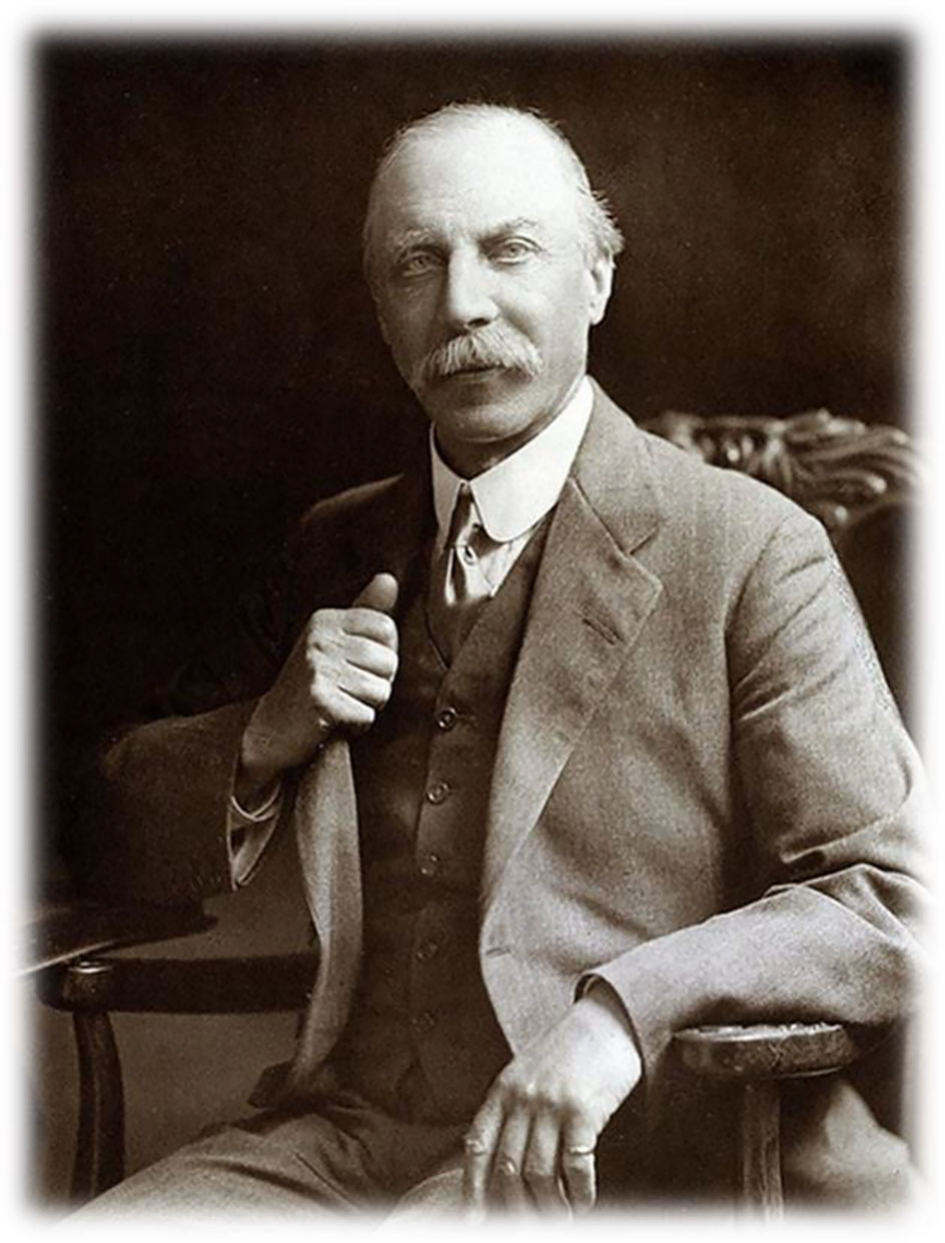
Sir Edward A. Sharpey‐Schafer (1850–1935). Editor‐in‐Chief of *Experimental Physiology* between 1908 and 1933. Copyright Lafayette Ltd, reproduced with permission Wellcome Collection.

By the time Sharpey‐Schafer founded *Experimental Physiology*, he had served on the Editorial Board of *The Journal of Physiology* for nearly 15 years. However, his influence was limited by the editorial suppression of John Newport Langley (1852–1925), whose authoritarian approach was well known and a source of considerable frustration within the scientific community (Whitteridge, 1983). At this stage, *the dissatisfaction with Langley's autocratic leadership of The Journal of Physiology* grew, prompting many to look to Sharpey‐Schafer for an alternative journal, with the enthusiastic encouragement of his friend, Sir Charles Scott Sherrington (1857–1952) (Bailey, Berg et al., 2023).

By Christmas 1907, Sharpey‐Schafer embarked on a mission to establish a new physiology journal with the collective backing of several members of The Physiological Society. Consequently, on Christmas Day, Sharpey‐Schafer issued a further circular to members of The Physiological Society, formally announcing the launch of the newjournal tentatively entitled, *Quarterly Journal of Physiology*. This provoked strong opposition from Langley, who argued that there was no need for a second British physiology journal, as it would only dilute research quality and impede the advancement of the discipline. As a consequence, Langley chose to quickly expel Sharpey‐Schafer from the Editorial Board of *The Journal of Physiology*.

An additional aspect that undoubtedly contributed to Langley's reservations was Sharpey‐Schafer's initial choice of title for the new journal, which Langley believed inappropriately associated with *The Journal of Physiology*. To address this concern, Sharpey‐Schafer (reluctantly) sent Langley a list of alternative titles, one of which proposed adding the word ‘experimental’ (Whitteridge, 1983). He also reached out to Ernest H. Starling (1866–1927), his former mentee and rising star of the time, who maintained good relations with both men. However, as Starling was in New York delivering the Harvey Lectures, it was his wife, Florence Starling, who responded. She had also received a letter on the same matter from Langley addressed to her husband. She diplomatically wrote to Sharpey‐Schafer (January 12^th^), looking to find the intellectual middle‐ground, striking a non‐confrontational compromise: ‘I feel quite sure my husband would prefer the title *Quarterly Journal of Experimental Physiology*. Since Professor Langley has brought *The* *Journal of Physiology* to its high state of efficiency, of which I have often heard my husband speak with appreciation, it would seem only just to honour his wishes in this regard’ (Henriksen, 2000). Despite the tensions between Sharpey‐Schafer and Langley, they maintained a healthy relationship of mutual admiration and respect. Thus, when Sharpey‐Schafer undertook the task of writing the *History of* *the Physiological Society During its First Fifty Years*, he paid tribute to the recently deceased Langley, writing: ‘Suffice it to say that from its formation in 1876 until his death, there was no more active participator in the work of The Physiological Society than John Newport Langley’ (Sharpey‐Schafer, 1927).

The inclusion of ‘experimental’ in the title of our journal after Florence Starling's measured input was neither arbitrary nor incidental. *Experimental Physiology* – the journal's current name since 1990 – was also the title of the practical laboratory course that Sharpey‐Schafer introduced during his tenure as Chair of Physiology at the University of Edinburgh. This course, which emphasised formal experimentation to illustrate various physiological principles and phenomena, was accompanied by a textbook of the same name, first published in 1912 (Schäfer, 1912). As such, the term ‘experimental’ refers to the philosophical basis of the field. The medical historian Andrew Cunningham, while examining the evolution of physiological discoveries from antiquity to the early 20th century, identified a fundamental distinction between anatomy and physiology before the early 19th century (Cunningham, 2002, 2003): while anatomists conducted dissections, physiologists engaged in speculation. He described physiology as ‘the speculative wing of anatomy’, distinguishing what he termed ‘old physiology’, which existed in two forms – natural philosophy and theoretical medicine.

Prior to the early 19th century, natural philosophers primarily sought to answer the question, ‘What is life?’, developing their understanding of organ function through observation, reasoning and speculation. Although their inquiries increasingly focused on life processes in animals, they remained largely dependent on anatomical studies and theoretical discourse rather than experimentation. However, by the early 19th century, physiologists including Claude Bernard (1813–1878), Carl Ludwig (1816–1896) and Eduard Friedrich Wilhelm Pflüger (1829–1910) began to address these questions through experimental methods, including live animal research and the systematic study of organ systems under induced perturbations. This paradigm shift marked the advent of a ‘new’ physiology – one rooted in empirical investigation and material experimentation, forming the foundation of the discipline as it stands today. Cunningham encapsulated this transformation with his well‐known metaphor of the pen and the sword: whereas ‘old physiology’ had been defined by theoretical inquiry (‘the pen’), ‘new physiology’ was shaped by experimental investigation (‘the sword’) (Cunningham, 2002, 2003). This metaphorical *jeu de mot* formed the conceptual basis of our previous editorial (Bailey, Berg et al., 2023).

With the *Quarterly Journal of Experimental Physiology*, Sharpey‐Schafer established a journal that, like *The Journal of Physiology* and The Physiological Society, was firmly rooted in the ‘new’ physiology as defined by Cunningham. Sharpey‐Schafer's philosophy as a scientist, teacher and editor held that experimentally based physiology was a fundamental prerequisite for the practice of clinical medicine (Schäfer, 1885, 1903). Moreover, while he maintained that systematic laboratory experimentation was essential for gaining direct mechanistic insights, he had the strategic foresight to adopt a broader definition of the field in his journal, incorporating related disciplines that encompassed biological chemistry, immunology and histology. This spirit endures to the present day, as *Experimental Physiology* continues to focus on studies that provide both mechanistic and translational insights taking advantage of a multidisciplinary integrative approach.

Beyond traditional laboratory‐based, applied and clinical studies, which have long been central to physiological research, the journal has adapted to the evolving landscape of biomedical sciences. Accordingly, *Experimental Physiology* also embraces population‐based prospective and retrospective cohort studies, as well as both observational and interventional clinical–surgical research focused on treatment effects – provided they offer insight into bodily functions and adaptations to environmental, pathological, behavioural or pharmacological stressors. A key ambition in this context is to further strengthen our clinical translational focus while bridging the gap between model/laboratory‐based and applied and environmental/space physiology in humans. The journal also encourages submission of methodological and diagnostic studies. Although these may not always adhere to a classical laboratory‐based experimental approach, they nonetheless align with the journal's titular experimental focus, as they represent advancements within the ‘new’ empirically and materially grounded physiology (Berg et al., 2025). Strategic appointments to our Editorial Board while also looking to improve equality, diversity and inclusion, reflect our collective ambitions to broaden our approach and encompass new and exciting areas of physiology, reaching out across the globe (Figure 2).

**FIGURE 2:**
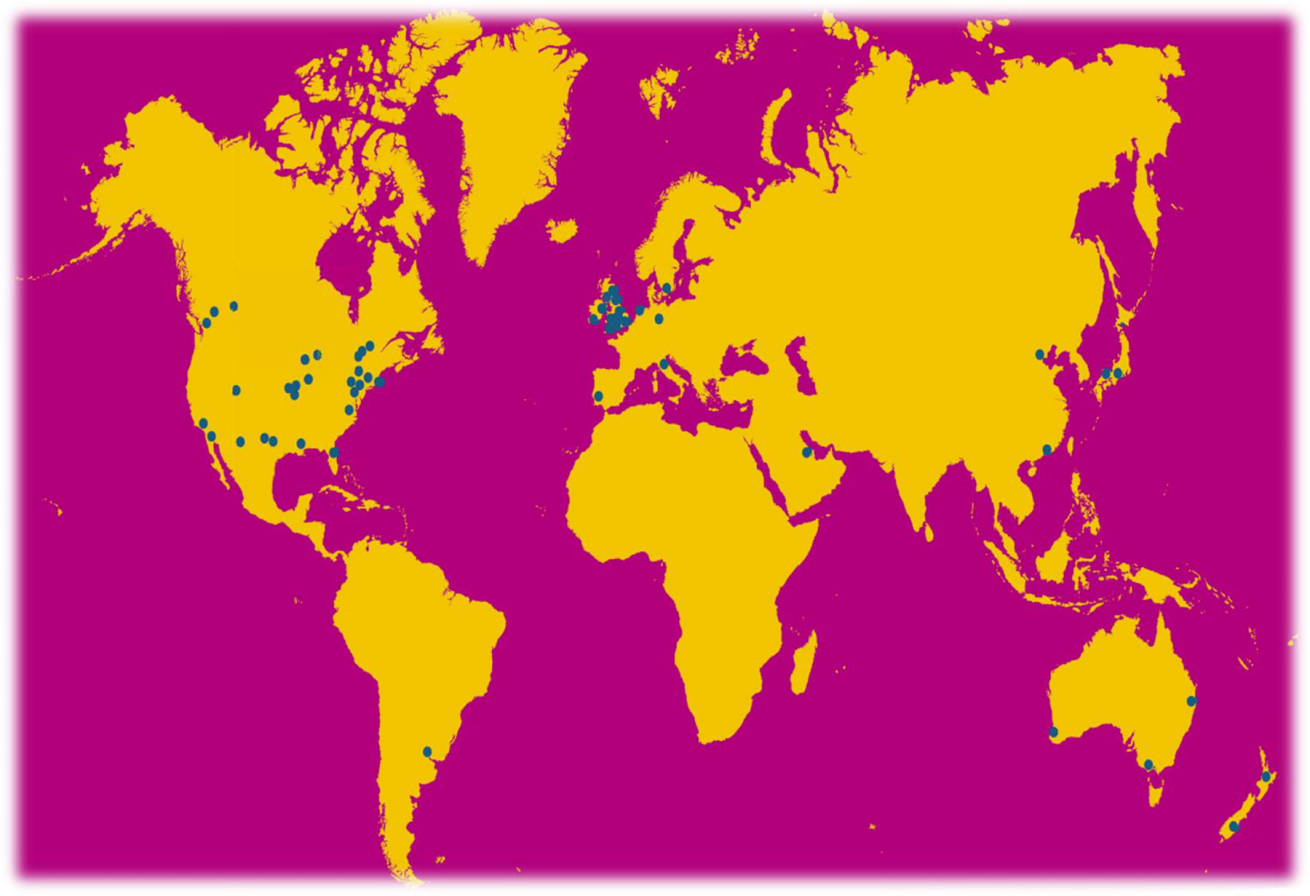
Geographical distribution of *Experimental Physiology*’s Editorial Board. Information provided by The Physiological Society (5 March 2025).

A related strategic initiative at *Experimental Physiology* involves the commissioning of targeted submissions to a burgeoning Special Issue ‘pipeline’, allowing the journal to publish issues dedicated to contemporary topics. The journal recently closed calls for a good number of such topics including: ‘The unspecific control of cardiac output during exercise and in (patho‐) physiology’; ‘Dietary manipulations for health and in the prevention and management of disease’; ‘Space physiology: challenges and solutions for a journey to Mars’; ‘Physiology and the Olympics’; ‘Exercise as medicine’; and ‘Mechanotransduction, muscle spindles and proprioception’. At the time of writing, calls for submissions remain open for: ‘The cellular/molecular mechanisms and potential treatment strategies for kidney diseases’; ‘New perspectives on the physiological basis of muscle loss’; ‘Integrative regulation of cerebral blood flow in response to exercise and environmental extremes’; ‘New approaches for old diseases’; and ‘Thermal physiology in health and disease: mechanisms and therapeutic applications’.

There will be many more special issue calls to come as we look to encourage more ideas and activity from our Editorial Board that includes the recent launch of our Future Leaders Scheme. The Future Leaders will join our Editorial Board for a period of two years and implement their Special Issue proposals (as part of the selection criteria) under the close mentorship of selected members of our Senior Editorial Team. Upon successful completion of their term, our ‘Future Leaders’ will be invited to join the Editorial Board as a Senior Editor. The future of physiology is (and will continue to be) bright! So, if you have any ‘hot’ topics of burning interest that you think will resonate with our readership, please complete the online *Experimental Physiology* special issue proposal form (https://www.physoc.org/journals‐and‐media/journals/special‐issue‐proposal‐form‐ep/) and reach out to our team with your ideas (ephjournal@physoc.org). Our publications team provides prospective authors with excellent guidance and support, which includes targeted commissioning tools offered by our publisher, Wiley, and it is a great way to organise and collaborate with peers to showcase the latest developments in our research fields. And an important point especially for early career physiologists, articles published in special issues are read and cited ∼20% more than those published in regular issues – visibility has become a key impact metric!

Our historical foundation within ‘new’ physiology as defined by Cunningham is further reflected in our growing emphasis on discursive content that explores scientific developments within a broader framework. This includes discussions on the fundamental principles of our discipline, their ethical and philosophical underpinnings and implications (Bailey & Poole, 2022; Berg & Bailey, 2024; Berg, Hamilton et al., 2024, Berg et al., 2025; Drummond & Tipton, 2024; O'Halloran, 2025; Poole & Bailey, 2024; Zacho, 2025), and methodological considerations, such as physiological measurement methodology, statistical matters, and transparency in reporting (Bailey, Rose et al., 2023; Berg, Christensen et al., 2024; Christensen et al., 2024; Hartmann et al., 2023; Rasmussen et al., 2024). With the latter in mind, we recently celebrated publication of our first Registered Reports in Physiology, which include both the Protocol and Results components (Rasmussen et al., 2024). Conceptually, registered reports differ from traditional scientific reporting, differentiated by the integration of peer review before data collection physically begins, and it offers unique benefits to our authors. This category, the first of its kind in our discipline, is testament to our commitment to improve how research is conducted, assessed, reported and incentivised. We recently released a video on the Registered Reports concept, to help put this publications category into clearer perspective (Rasmussen et al., 2025).

These themes are also highlighted in our popular Methods & Techniques (formerly Myths & Methodologies) series, with recent papers on methods for assessing respiratory muscle activity in humans (Hudson et al., 2025), methods for assessing glycaemic control (Wrench et al., 2014), thermoregulatory adaptations during heat acclimatisation (Tyler & Notley, 2024), and standardisation in human experimental physiology research (Merrell et al., 2024), and the validity and reliability (or lack thereof!) of the so‐called mean flow index to assess dynamic cerebral autoregulation (Olsen et al., 2024), to mention a select few. They are also reflected in our expanded focus on the history of physiology *à la* Confucius (551–479 B.C.E.), ‘Study the past if you would define the future’, encompassing The Physiological Society, and its expanding family of journals (see later). Recent examples of this commitment include several articles on the history of the Scandinavian exercise physiology tradition (Aalkjaer et al., 2023; Berg, 2024a, Berg, Hamilton et al., 2024; Berg et al., 2025), alongside The Physiological Society's newly launched Blue Plaque Series, which highlights and examines the seminal contributions of scientists honoured through The Physiological Society's Blue Plaque initiative; for instance, the recently published Blue Plaque reviews on the physiology icon and founding father of exercise physiology, A. V. Hill (Burnley et al., 2025) and M.P. Fitzgerald, the first centenarian to receive an honorary degree from the University of Oxford (Hudson et al. 2025).

Another key aspect of Sharpey‐Schafer's editorial philosophy was ensuring that papers reflected the author's unbridled, personal voice. This was in riposte to Langley's ruthlessly constrained editing of papers published in *The Journal of Physiology*, a process that some of its ‘victims’ referred to as being ‘Langleyized’ (Whitteridge, 1983). However, Langley's approach to scientific publishing – maintaining high standards of form and style, ensuring clarity, conciseness, and minimal speculative discussion – has stood the test of time and has profoundly influenced scientific writing across a multitude of biomedical disciplines (Berg, Hamilton et al., 2024). Nonetheless, beyond the systematic and unbiased presentation of findings, preserving the author's voice to help humanise our science remains a fundamental principle in *Experimental Physiology*. This is reflected in various ways, including commissioned articles that allow scientists to share their personal narratives – ranging from the experiences (trials and tribulations) of early career researchers as part of a growing collection (Burma, 2025; Kaur, 2025; Yfanti, 2025), to reflections of established scientists’ careers and major discoveries (Aalkjaer et al., 2023; Bärtsch, 2025; Brassard, 2024; Montgomery, 2025). Additionally, the ‘Physiology of Lived Experience’ series, conceived by our former Editor‐in‐Chief and President‐Elect to The Physiological Society, Professor Mike Tipton, explores how knowledge of integrative physiology can both inform and be informed by one's personal experience (Tipton, 2025). These editorials will help encourage discourse and bring out the fun of physiology, a key ingredient that should be encouraged across the continuum of all physiologists’ career stages.

Recent audit of *Experimental Physiology*’s performance reveals that our growth is positive, which is encouraging as our (inevitable) recovery post COVID‐19 pandemic, and flip to Open Access in January 2023, continues. The number of acceptances in 2024 have exceeded those reported in 2023 as we look to continue to forge this upward trajectory into 2025 (Figure 3). We can attribute our growth to a number of strategic changes that include: increased volume of transfers from *The Journal of Physiology* (Figure 3, c/o Editor‐in‐Chief Professor Kim Barrett, to whom we are indebted); change in our editorial strategy to work more actively with authors to help them achieve the ‘quality bar’ required for publication; and finally, a higher proportion of commissioned content via our Special Issue pipeline. Figure 4 illustrates select examples of some of the strategic changes that our Senior Editorial Team has wrestled with over the years, and that has been a joy to be involved with. They are living examples of ‘swordsmen and swordswomen’ of modern physiology (Bailey, 2022; Bailey & Stewart, 2022)!

**FIGURE 3:**
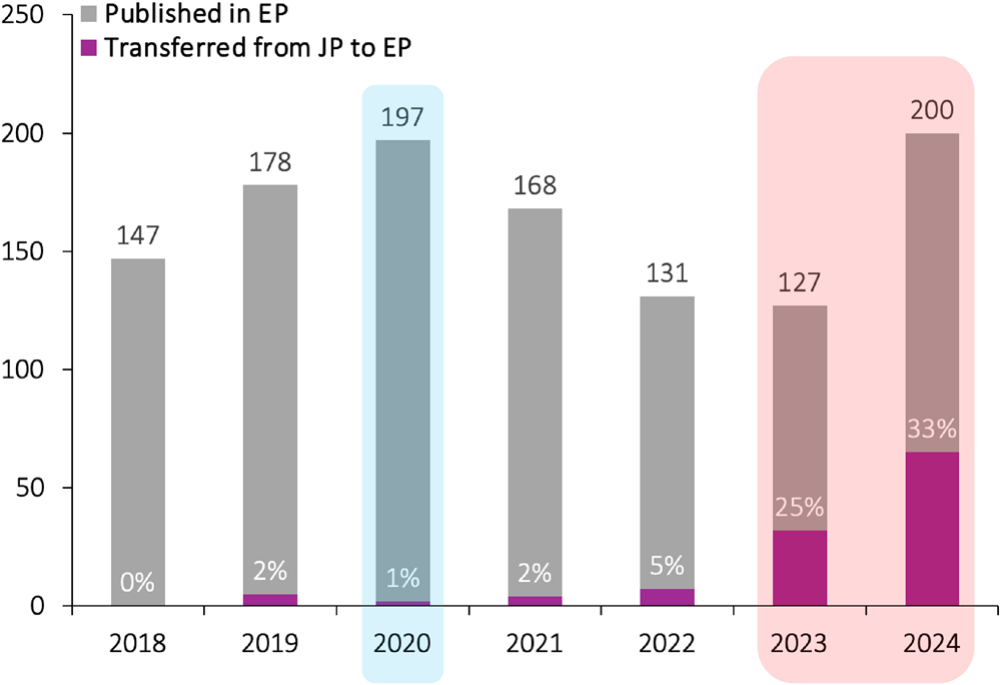
Select performance metrics for *Experimental Physiology*. Published articles in *Experimental Physiology* include Research Articles, Short Communications, Case Reports, Methods & Techniques, Review Articles and Lectures. Blue shading refers to start of COVID‐19 lockdown. Red shading reflects the period in which *Experimental Physiology* transitioned to Open Access. Note the (papers) transferred from *Journal of Physiology* (JP) to *Experimental Physiology* (EP) are represented in absolute terms (bars) and expressed as a percentage of the total number of papers published in EP. Information provided by The Physiological Society (27 March 2025).

**FIGURE 4:**
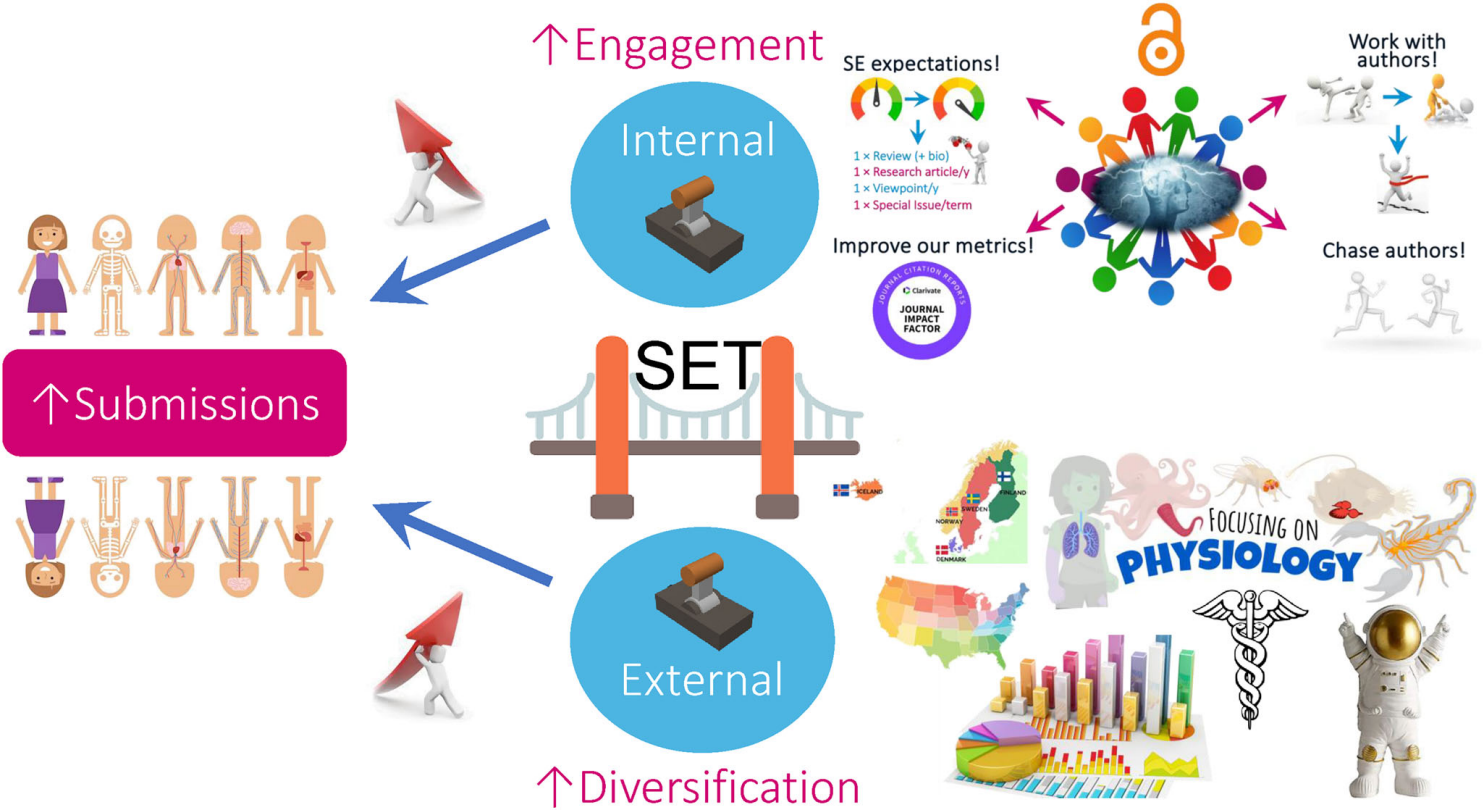
Strategic changes to *Experimental Physiology*. Implementation of our Senior Editorial Team (SET) has sought to ‘bridge the gap’ and improve internal and external engagement with our journal. This has taken the form of increasing Editorial Board engagement with clear expectations of our Senior Editors, expanding our Special Issues pipeline and changing the way we work with our authors (↑engagement). We have also expanded Editorial Board membership to reflect a wider geographical and more contemporary focus (↑diversification).

We are also keen to publish guidelines, position stands and white papers in the true collaborative spirit of integrative multidisciplinary science that will help improve our impact factor, that key albeit controversial metric that invariably influences journal choice especially among early career researchers. We aspire to *Experimental Physiology* becoming that journal ‘of choice’! And on a collaborative note, we are delighted that The Physiological Society's ‘family’ of journals has expanded with the recent launch of *The Journal of Precision Medicine: Health and Disease* and *The Journal of Nutritional Physiology*, led by our inaugural Editors‐in‐Chief, Professors Colleen Clancy and Craig Sale, respectively (Figure 5). These new journals have been selected to complement and not compete against our existing journals and will bring additional exciting opportunities to the wider physiological community. Building on strategic principles laid out at the very beginning of our tenure(s) (Bailey, Berg et al., 2023), we are looking forward to working with (and not against, as chequered times past!) one another and exploring collaborative opportunities while benefitting from cross‐journal referrals/transfers, to better harness the collective ‘power of physiology’ (Bailey, 2022; Bailey & Stewart, 2022) – *unus pro omnibus, omnes pro uno*!

**FIGURE 5:**
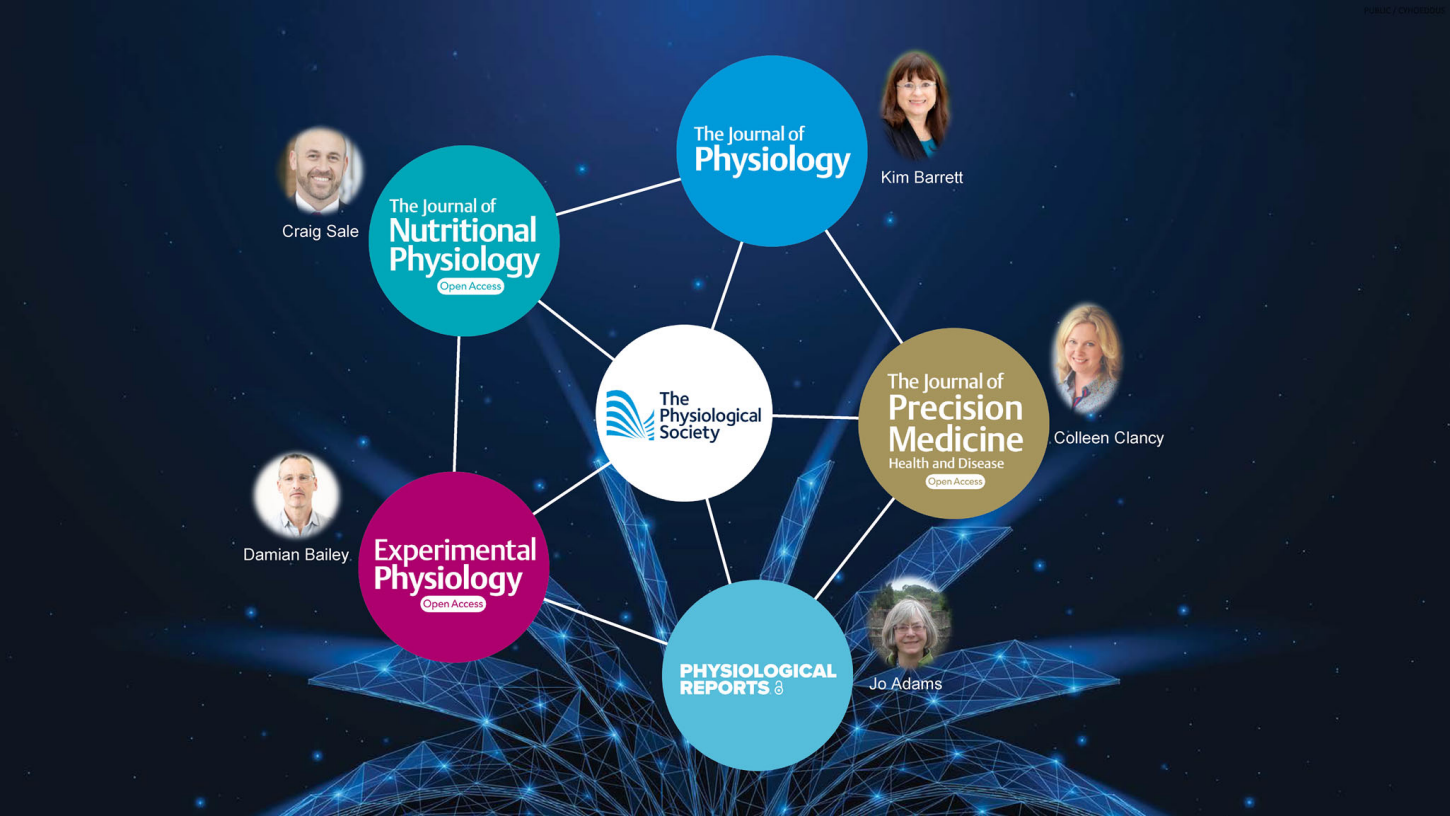
Family of journals and current Editors‐in‐Chief under the auspices of The Physiological Society. Reproduced with permission The Physiological Society.

And thus, to clarify in modern times, physiology remains an inherently experimental science closely linked to clinical medicine—an unabashed truth proudly reflected in our journal’s title and integrative‐translational scope. *Experimental Physiology* can look forward to a bright future, supported by a world‐class Editorial Board and outstanding Editorial Team. As an official journal of The Physiological Society, we also aim to inspire and encourage Society members to become more actively involved for the benefit of our collective discipline – whether as authors, referees or editors. *In nuce*, ‘Support Society journals – because they support you!’ – a veritable call to arms!

## AUTHOR CONTRIBUTIONS

D.M.B. and R.M.G.B. conceived the idea and wrote the first draft of the manuscript. D.C.P. edited and revised the manuscript. D.M.B., D.C.P. and R.M.G.B. approved the final version submitted for publication and agree to be accountable for all aspects of the work in ensuring that questions related to the accuracy or integrity of any part of the work are appropriately investigated and resolved. All persons designated as authors qualify for authorship, and all those who qualify for authorship are listed.

## CONFLICT OF INTEREST

D.M.B. is Editor‐in‐Chief of *Experimental Physiology*, Chair of the Life Sciences Working Group, member of the Human Spaceflight and Exploration Science Advisory Committee to the European Space Agency and member of the Space Exploration Advisory Committee to the UK and Swedish National Space Agencies. D.M.B. is also affiliated to Bexorg, Inc. (USA) focused on the technological development of novel biomarkers of cerebral bioenergetic function and structural damage in humans. D.C.P. is Deputy Editor‐in‐Chief (USA) of *Experimental Physiology*. R.M.G.B. is Deputy Editor‐in‐Chief (Europe) of *Experimental Physiology*. D.M.B., D.C.P. and R.M.G.B. were blinded from the review process and from making any editorial decisions for this manuscript.
